# DISRUPTED SOCIAL LIFE MEDIATES ASSOCIATION BETWEEN PANDEMIC WORRIES AND RISK OF ELDER ABUSE

**DOI:** 10.1093/geroni/igad104.3284

**Published:** 2023-12-21

**Authors:** Louis To, Lok Man Leung, Debby Wan, Ronald Tsui, Elsie Yan

**Affiliations:** The Hong Kong Polytechnic University, Hong Kong, Hong Kong; The Hong Kong Polytechnic University, Hong Kong, Hong Kong; The Hong Kong Polytechnic University, Hong Kong, Hong Kong; The Hong Kong Polytechnic University, Hong Kong, Hong Kong; The Hong Kong Polytechnic University, Hong Kong, Hong Kong

## Abstract

Social isolation is an important risk factor for elder abuse. This study investigated the mediating effects of pandemic-related disruptions of social life between worry about the pandemic and risk of elder abuse. A total of 1078 community dwelling older persons participated in this community survey. They provided information on their demographics, worry about the pandemic, pandemic related disruptions of social life, and risk of elder abuse as measured by a 10-item scale derived from Dong et al. (2015). Participants age ranged from 60 to 100 (mean= 76.2, SD=7.9), majority of them were female (81.6%), single, divorced or widowed (52.8%). Prevalence of risk of abuse was high (21.5%), with the most prevalent type being family conflicts at home (16.6%), followed by feeling uncomfortable with someone in the family (8.1%). Participants who were not in a marital relationship, of larger household, reported feeling worried about the pandemic and felt their social life disrupted were at greater risk of abuse. Logistic regression results show that pandemic related disruptions of social life partially mediated the association between worry about the pandemic and risk of abuse. In the final model, participants who reported feeling worry about the pandemic and those who reported that the pandemic has caused disruptions in their social life were at greater odds of reporting risk of abuse (OR=1.14 and 1.13 respectively, p<.05). The effects of the former diminished after the latter was entered into the model. This finding highlights the importance of social life in protecting older persons against abuse.

